# Transcriptional Activity in Diplotene Larch Microsporocytes, with Emphasis on the Diffuse Stage

**DOI:** 10.1371/journal.pone.0117337

**Published:** 2015-02-11

**Authors:** Agnieszka Kołowerzo-Lubnau, Janusz Niedojadło, Michał Świdziński, Elżbieta Bednarska-Kozakiewicz, Dariusz J. Smoliński

**Affiliations:** 1 Department of Cell Biology, Faculty of Biology and Environment Protection, Nicolaus Copernicus University, Toruń, Poland; 2 Centre For Modern Interdisciplinary Technologies, Nicolaus Copernicus University, Toruń, Poland; International Centre for Genetic Engineering and Biotechnology, ITALY

## Abstract

Manuscript provides insights into the biology of long-lived plants, different from Arabidopsis, tomato or grass species that are widely studied. In the European larch the diplotene stage lasts approximately 5 months and it is possible to divide it into several substages and to observe each of them in details. The diplotene stage is a period of intensive microsporocyte growth associated with the synthesis and accumulation of different RNA and proteins. Larch microsporocytes display changes in chromatin morphology during this stage, alternating between 4 short stages of chromatin condensation (contraction) and 5 longer diffusion (relaxation) stages. The occurrence of a diplotene diffusion stage has been observed in many plant species. Interestingly, they have also been observed during spermiogenesis and oogenesis in animals. The aim of this study was to examine whether chromatin relaxation during the diplotene is accompanied by the synthesis and maturation of mRNA. The results reveal a correlation between the diffusion and chromatin decondensation, transcriptional activity. We also found decreasing amount of poly(A) mRNA synthesis in the consecutive diffusion stages. During the early diffusion stages, mRNA is intensively synthesized. In the nuclei large amounts of RNA polymerase II, and high levels of snRNPs were observed. In the late diffusion stages, the synthesized mRNA is not directly subjected to translation but it is stored in the nucleus, and later transported to the cytoplasm and translated. In the last diffusion stage, the level of poly(A) RNA is low, but that of splicing factors is still high. It appears that the mRNA synthesized in early stages is used during the diplotene stage and is not transmitted to dyad and tetrads. In contrast, splicing factors accumulate and are most likely transmitted to the dyad and tetrads, where they are used after the resumption of intense transcription. Similar meiotic process were observed during oogenesis in animals. This indicates the existence of an evolutionarily conserved mechanism of chromatin-based regulation of gene expression during meiotic prophase I.

## Introduction

Meiosis is an important step in sexual reproduction. It is a specialised cell division that generates four haploid cells from a diploid parent cell after a single round of DNA replication and two consecutive rounds of nuclear division. In the first nuclear division, homologous chromosomes segregate, and in the second division, sister chromatids segregate. Accurate chromosome segregation during meiosis is essential for haploid gamete formation [1]. The most complex phase of meiotic division is meiotic prophase I. During this tage, nuclear organisation dramatically changes. Early meiosis stages are associated with a meiosis-specific organisational structure known as synaptonemal complex (SC) formation, pairing and recombination between homologous chromosomes. However, few details of the processes that occurafter recombination are known. The diplotene stage lasts for approximately 4–5 months in various larch species, and it includes several chromatin relaxation stages. These “diffuse stages,” which occur in larch microsporocytes in the late fall and early winter, were previously considered resting stages resulting from low temperatures [2,3,4]. The occurrence of a diffusion stage has been observed in many plant species [5–13].However, recent studies in *L*. *leptolepis* microsporocytes have shown that chromosomes undergo several gradual and major organisational changes during the diplotene stage [14]. Interestingly, the diffusion phase during diplotene has also been observed during spermiogenesis and oogenesis in animals [15–19].

According to the proposed model [20], chromosomes undergo an organisational change from meiotic to interphase during the diplotene stage and the diffusion stage is analogous to the G2 stage of interphase. This chromatin reorganisation induces the chromosomes to become transcriptionally active [8,21,22]. The reported results [14] are consistent with this model. These data do not support the hypothesis that the diffusion stage is a transcriptionally silent period. There have not been any studies that examine the level and distribution of various types of RNA in the diplotene stage; thus, the present study aimed to examine the patterns of RNA synthesis and distribution in European larch microsporocytes during the diplotene stage. In addition, previous studies of the diplotene stage have revealed the synthesis of large quantities of proteins [23]. Therefore, we comprehensively examined the transcriptional activity, levels and distribution of poly(A) RNA, RNA polymerase II (an enzyme responsible for mRNA synthesis) and snRNAs, which are responsible for mRNA maturation. We also examined whether mRNA synthesis and maturation is accompanied by protein synthesis.

## Materials and Methods

### Plant material and isolation of meiotic protoplasts

The study was carried out in the botanical garden of our home Faculty Biology and Environmental Protection of Nicolaus Copernicus University, no specific additional permissions were required. To maintain consistent experimental conditions, *Larix decidua* Mill. anthers were collected from the same tree in successive meiotic prophase stages of the diplotene stage from November until March at weekly intervals. Anthers were fixed in 4% paraformaldehyde in phosphate-buffered saline (PBS), pH 7.2, for 12 h and squashed to obtain free meiocytes. Meiotic protoplasts were isolated from these cells [24] and were then subjected to immunodetection of newly formed transcripts (BrU—RNA), detection of protein synthesis, RNA polymerase II (RNA pol II) and m3G snRNA and detection of poly(A) RNA and U2 snRNA via fluorescence *in situ* hybridization (FISH).

For transmission electron microscopy analyses, cells were fixed in 4% paraformaldehyde and 0.25% GA in Pipes buffer overnight at room temperature, dehydrated in alcohol and embedded in LR Gold resin (Sigma, St. Louis, Mo., USA). The resultant material was sectioned using a Leica Ultracut UCT Ultramicrotome, and the sections were placed on nickel-formvar-coated grids.

### Design of double-labeling reactions

Several double-labeling immunofluorescence-FISH reactions (bromouridine (BrU): poly(A) RNA; m3G snRNA: U2 snRNA) and double immunofluorescent labeling (bromouridine (BrU): RNA polymerase II) were performed as described below. In the double-labeling immunofluorescence-FISH reactions, the immunocytochemical methods always preceded the *in situ* hybridization methods because when *in situ* hybridization was applied first, the subsequent levels of immunofluorescence signals were very weak. In the double immunofluorescence labeling experiments with bromouridine and polymerase II RNA labeling, BrU labeling always preceded RNA pol II labeling because the secondary anti-mouse IgM antibody used to detect the anti-RNA pol II primary antibody can react with both the IgM and IgG primary antibodies, whereas the anti-mouse IgG antibody does not react with the primary IgM antibody and only binds the primary IgG antibody.

### Bromouridine incorporation and immunodetection of newly formed transcript

Immunodetection of bromouridine incorporation prior to FISH or immunofluorescence staining in double-labeling reactions. Bromouridine incorporation was accomplished according to the method [25], with a long (90 min) incubation time. In the immunolabeling experiments, the protoplasts were then incubated with the primary mouse anti-BrU antibody (F. Hoffmann-LaRoche Ltd., Rotkreuz, Switzerland) in PBS with 1% BSA, pH 7.2 (diluted 1:100), overnight at 4°C, followed by a goat anti-mouse secondary antibody (IgG) conjugated with Alexa Fluor 488 in PBS with 0.2% BSA (diluted 1:500) for 1 h at 37°C. The protoplasts were next washed in PBS, and the FISH poly(A), rRNA or RNA pol II detection method was applied. The samples were then washed in PBS. Finally, the slides were stained using DAPI (1 μg/ml for 3 min), washed in double-distilled water, and mounted in ProLong Gold antifade solution (Invitrogen).

### FISH detection of mature mRNA, maturation factor U2 snRNA and rRNA

For the hybridization assays, the probes were resuspended in hybridization buffer (30% v/v formamide, 4× SSC, 5× Denhardt’s buffer, 1 mM EDTA, 50 mM phosphate buffer) at a concentration of 50 pmol/ml, and hybridization was performed overnight at 37°C. The following antisense DNA oligonucleotides were used in the reactions of the detection of poly (A) RNA and U2 snRNA: poly(A) RNA—5′ Alexa 594 T(T)29 3′, U2 snRNA—5′ Alexa 594 ATATTAAACTGATAAGAA CAGATACTACACTTG 3′ (Genomed, Warsaw, Poland, and Sigma Proligo, USA), ITS1a—5′ Cy3 TCTTGCAAATCAACAGCCAC 3′, ITS1b—5′ Cy3 ATTATAGCAAGAGCATAACAAGCACAC 3′. The following sense DNA oligonucleotides were used in the control reactions: 5′ Alexa 594 A(A)29 3′, 5′ Alexa 594 CAAGTGTAGTATCTGTTCTTATCAGTTTAATAT 3′, 5′ Cy3 GTCGTGTT GATTTGCAAGA 3′, 5′ Cy3 GTGTGCTTGTTATGCTCTTGCTATAAT 3′ (Genomed, Warsaw, Poland, and Sigma Proligo, USA).

### Immunofluorescence detection of mature splicing snRNA and RNA polymerase II enzyme

Immunofluorescence methods for double-labeling reactions. Assays involving the m3G cap–anti-m3G antibody (Calbiochem, Bad Soden, Germany) were performed as [26]. RNA pol II was labeled with two monoclonal mouse antibodies (IgM): H14, which recognizes the hyperphosphorylated Ser-5 moiety in CTD repeats and preferentially recognizes the form of RNA pol II that is competent for the initiation of transcription, and H5, which recognizes the Ser-2-phosphorylated form of RNA pol II, which plays an essential role in transcriptional elongation (Agrisera AB, Vannas, Sweden). We confirmed the specificity of these antibodies in plant material in a previous immunoblot analysis [27]. In immunolabeling experiments, protoplasts were incubated with a primary mouse H14 or H5 antibody in PBS with 1% BSA, pH 7.2 (diluted 1:300), overnight at 4°C, followed by a goat anti-mouse secondary antibody (IgM) conjugated with TRITC in 0.2% BSA in PBS (diluted 1:50) for 1 h at 37°C. The samples were then washed in PBS. Finally, the slides were stained using DAPI (1 μg/ml for 3 min), washed in double-distilled water, and mounted in ProLong Gold antifade solution (Invitrogen).

### DNA detection (the Feulgen procedure)

After fixation in Carnoy’s solution (60% ethanol, 30% chloroform and 10% glacial acetic acid), anthers were washed in 95% ethanol and incubated with 1 N HCl at 60°C for 10 minutes. Next, the anthers were washed with water and incubated in Schiff’s reagent for 1 h at room temperature in the dark, followed by rinsing in bisulfite solution (5 ml of 10% potassium metabisulfite, 5 ml of 1 N HCL, 90 ml of distilled water) in 3 washes of 5 min each and thorough rinsing with water. Then, smears were prepared on 2.5% gelatin-coated slides and frozen on dry ice. The slides were finally dehydrated in a 70%, 96% and 100% alcohol series for 2 min each, followed by mounting with Euparal (CARL ROTH GmbH, Karlsruhe, Germany) under coverslips.

### DNA detection at the EM level

For in situ detection of structures containing DNA at the ultrastructural level, immunolabeling with monoclonal mouse IgM anti-DNA antibodies (Roche, diluted 1:25) and anti-mouse IgM secondary antibodies with 10 and 20 nm gold particles (BioCell, Cardiff, diluted 1:30) were used. Grids were then rinsed and contrasted (1% phosphotungstic acid and 2.5% uranyl acetate).

### Quantitative measurements

Image analysis of the larch microsporocyte protoplasts was performed after immunofluorescence staining with antibodies against m3G snRNA and RNA Pol II and after *in situ* hybridization. Each reaction step was performed using consistent temperatures, incubation times and concentrations of probes and primary and secondary antibodies. A total of 35 cells from each stage were analyzed following 3 different preparations. Three-dimensional optical sections were acquired with a 0,5-μm step interval. For all antigens and developmental stages, the obtained data were corrected for background autofluorescence, as determined by negative control signal intensities. The Lucia General software (Laboratory Imaging, Prague, Czech Republic), was used for image processing and analysis which is compatible with the images collected in the Nikon PCM 2000 confocal microscope and Nikon C1 confocal microscope as well as other microscopes manufactured by Nikon. The Kruskal–Wallis ANOVA test was applied to test for differences between multiple samples (groups, i.e., signal levels in different stages).

For colocalization analysis before measurement we perform the proper controls, including specimens labeled with each probe alone as well as an unstained autofluorescence standard. At the beginning of the measurements we performed software correction of bleed-through before proceeding with colocalization analysis. We calculated the % of fluorescence intensity in the “newly formed RNA” channel covered by the colocalization area.

### Microscopy

The results were registered with Nikon C1, Nikon PCM 2000, Leica SP8 confocal microscopes using lasers emitting light at a wavelengths of 405, 488, 543, 561 and 594 nm. For Nikon confocal microscopes a 60X (numerical aperture, 1.4) Plan Apochromat DIC H oil immersion lens were used. Images were collected collected sequentially in the green (Alexa 488 fluorescence) and red (TRIC or Alexa 594) channels. To minimize bleed-through between fluorescence channels, we employed low laser power (3–10% of maximum power) and single-channel collection. For bleed-through analysis and control experiments, Lucia G software was used (Laboratory Imaging, Prague, Czech Republic). For Leica confocal microscope an optimized pinhole, long exposure time (400 kHZ) and 60X (numerical aperture, 1.4) Plan Apochromat DIC H oil immersion lens were used. Images were collected sequentially in the blue (DAPI), in the green (Alexa 488 fluorescence) and red (Alexa 594, Cy3) channels. To minimize bleed-through between fluorescence channels, we employed low laser power (0.4–5% of maximum power) and single-channel collection. For bleed-through analysis and control experiments, Leica SP8 software was used.

**Control reactions**. Control treatments consisting of incubations without the primary antibody were performed for the in situ hybridizations and immunofluorescence assays. For the in situ hybridization, high-resolution and fluorescence analyses, sense-labeled probes and ribonuclease-treated samples were used as additional controls. For the detection of new transcripts, a control reaction was performed in the protoplasts without incubation with bromouridine. All control reactions either produced negative results or results that were very weak compared with the standard reactions (S5 Fig.).

## Results

### Morphological changes in chromatin during diplotene in larch microsporocytes

**Fig 1:**
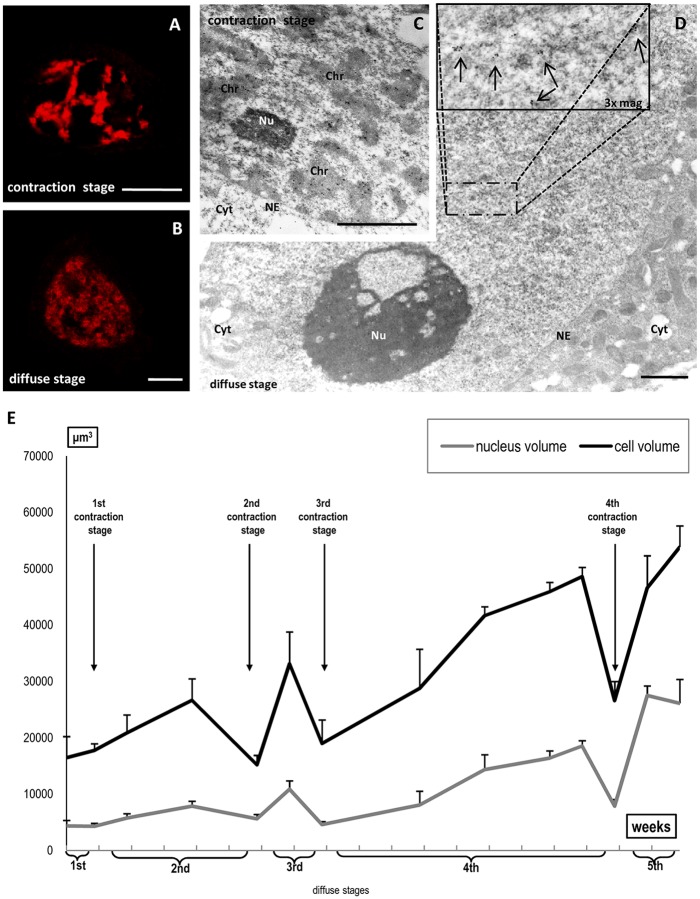
Examples of the two types of chromatin morphology. **A,B**—Feulgen staining. Bars—10 μm; Microsporocyte ultrastructure of contraction stage (**C**) and diffusion stage (**D**), anti-DNA localisation at the EM level. Strong and specific labelling was observed within condensed chromatin (**C**). Dispersed cluster labelling (arrows, **D**). Gold particles: **C**—20 nm, **D**—10 nm. Bars—1 μm (Chr—chromatin; Cyt—cytoplasm; NE—nuclear envelope; Nu—nucleolus). The nuclear and cellular volume during the subsequent diplotene stages **(E)**.

**Fig 2:**
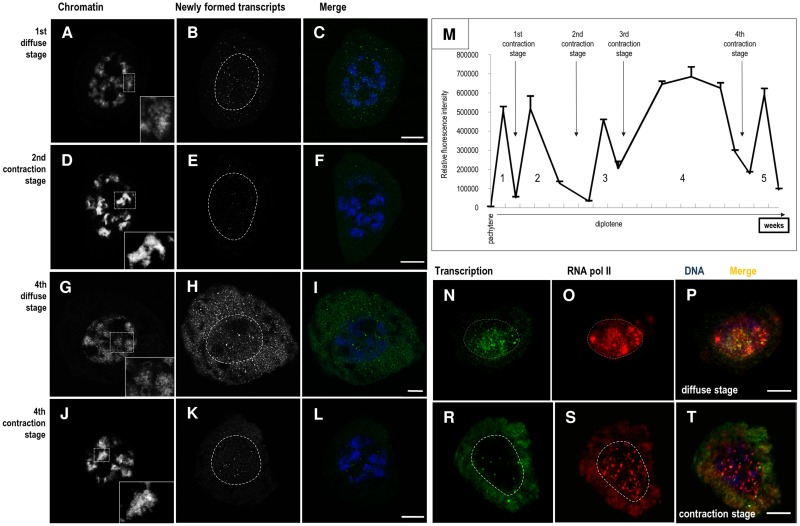
Chromatin morphology and transcriptional activity during microsporocyte development (A—L). Chromatin morphology detected via DAPI staining during the diffuse (**A**, **G**) and contraction (**D**, **J**) stages; Newly formed transcripts, detected via BrU incorporation, was performed with a long (90 min) incubation time in diffuse (**B**, **H**) and contraction (**E**, **K**) stages. **C, F**, **I**, **L**—merge. Bars—10 μm. **Total newly formed transcripts contents during microsporocyte development (M)**; 1–5—periods of high transcription activity correlated with diffuse stages. **Distribution of newly formed transcripts and active polymerase RNA II (N—T)**. **N—**distribution of newly formed transcript during diffuse stages; **O—**distribution of RNA pol II during diffuse stages using the H5 antibody, which recognises Ser-2-phosphorylated RNA pol II; **P—**merge. **R**—distribution of newly formed transcripts during chromatin condensation stages; **S**—distribution of RNA pol II during chromatin condensation stages (H5 antibody); **T**—merge. Bars—10 μm.

The diplotene stage in European larch microsporocytes begins in November and continues until March. We collected flower buds weekly (22 harvests) and obtained protoplasts. We prepared the cells and stained the DNA to measure the cell and nuclei volume. Our analysis indicates that 70–95% of the cells collected from one harvest were similar in size and chromatin morphology. The characteristic changes in chromatin morphology and cell size were accompanied by changes in the localisation and amount of several factors. These data allowed us to distinguish between 14 stages in the diplotene stage. We observed similar changes in cell size and morphology of the chromatin *in vivo* (cell/nuclear volume; chromatin morphology using the cell-permeable dye Hoechst 33342, which can bind to DNA in live cells) and after paraformaldehyde or Carnoy’s fixation. Strong chromatin condensation during the pachytene stage was followed by chromatin diffusion at the beginning of the diplotene stage, but we also observed 4 transient stages in which chromatin was more compact (contraction stages) that alternated with 4longer diffuse stages, although of various lengths (S1–4 Figs.). We distinguished successive diplotene substages by DAPI chromatin staining or Feulgen staining (Fig. 1A and 1B) and confocal analysis, which showed the characteristic morphological changes. Additionally, we generated 3D projection images (S1–4 Figs., left panel) from optical sections of representative cells at each sub stage (S1–4 Figs., right panel). To better determine the position of the nucleolus, the localisation of rRNA (red colour) is shown on the 3D images. For rRNA localisation, we performed in situ hybridisation with probes complementary to 26S rRNA or the ITS (internal transcribed spacer) sequence. Due to the very strong mature rRNA signal in the nucleolus and nucleoplasm during the first three diffuse stages, we imaged ITS localisation, which did not disturb the view of the chromatin structure. Diffuse stages were characterised by the presence of chromatin fibres (Fig. 1B), which often formed loops, especially in the middle diplotene stages (Fig. 2G and S3 Fig.). Ultrastructural DNA localisation revealed specific diffuse cluster labelling in the area surrounding dispersed chromatin (Fig. 1D). In turn, chromatin often appeared as dense patches staining in the contraction stages, which were clearly visible after Feulgen (Fig. 1A) and DAPI staining (Fig. 2D, 2J and S1–4 Figs.), as well as at the ultrastructural level (Fig. 1C).

During the 5-month-long diplotene stage, microsporocytes extensively grew, and their volume gradually increased. In the middle of the diplotene stage, the cells were 2-foldlarger, and by the end of diplotene, they were 3-fold larger than at the beginning of the diplotene stage. Cell growth was associated with nuclear volume increases (Fig. 1E). At the end of the diplotene stage, the nuclear volume was 6 times greater than at the beginning of the stage. However, despite the general trend of microsporocyte growth during the diplotene stage, the cell and nuclear volume decreased during certain stages (Fig. 1E). The contraction stage was accompanied by a reduction in cell under various fixation conditions and in *in vivo* studies (unpublished data). Therefore, our findings are representative of plasticity rather than an artefact of the procedures.

### Transcriptional activity is associated with chromatin morphology changes

We examined whether the changes in cell and nuclear volume and in chromatin morphology were associated with changes in microsporocyte transcriptional activity. Using the BrU incorporation technique, we examined the distribution patterns and level of newly formed transcripts in diplotene larch microsporocytes. Long incubations of cells with BrU (1,5 h) allowed us to visualise newly formed transcripts at the site of synthesis and their transportation into the cytoplasm. The results revealed that there was a correlation between chromatin morphology and RNA synthesis. In cells in which chromatin was organised into fibres, labelling of the newly formed transcript was much higher, and we observed signal in the nucleus and cytoplasm (Fig. 2B,2C,2H and 2I). Particularly high label levels characterised the cells in which chromatin fibres were numerous and very well distinguished (Fig. 2G,2H and 2I). In these stages, we observed BrU signal throughout the nucleoplasm in the form of very small clusters, where as these clusters were much more distinct and numerous in the cytoplasm (Fig. 2H). During stages where chromatin primarily formed dense patches, the level of newly formed transcript labelling was low (Fig. 2E,2F,2K and 2L). Quantitative studies showed that there were 5 periods of high transcriptional activity during the diplotene stage. The first stage took place in the late pachytene and early diplotene stage, and the other 4 occurred during the diplotene stage (Fig. 2M). These periods of high transcriptional activity corresponded to the diffuse chromatin stages. In contrast, the 4 periods of chromatin contraction corresponded to periods of decreased transcription (Fig. 2M).

### mRNA levels during successive diffuse and contraction stages

We examined whether the high transcription level was associated with mRNA synthesis. Through double labelling of newly formed transcripts, we analysed RNA polymerase II distribution, which is responsible for the synthesis of protein-encoding transcripts and most splicing snRNAs, in periods of high and low transcriptional activity. During the periods of high transcriptional activity (Fig. 2N), RNA polymerase II (Fig. 2O) was both dispersed and clustered, in which we observed its colocalisation with newly formed RNA (Fig. 2P). During the periods of reduced transcription (Fig. 2R), RNA polymerase II was only in clusters (Fig. 2S), often colocalising with newly formed transcripts (Fig. 2T).

**Fig 3:**
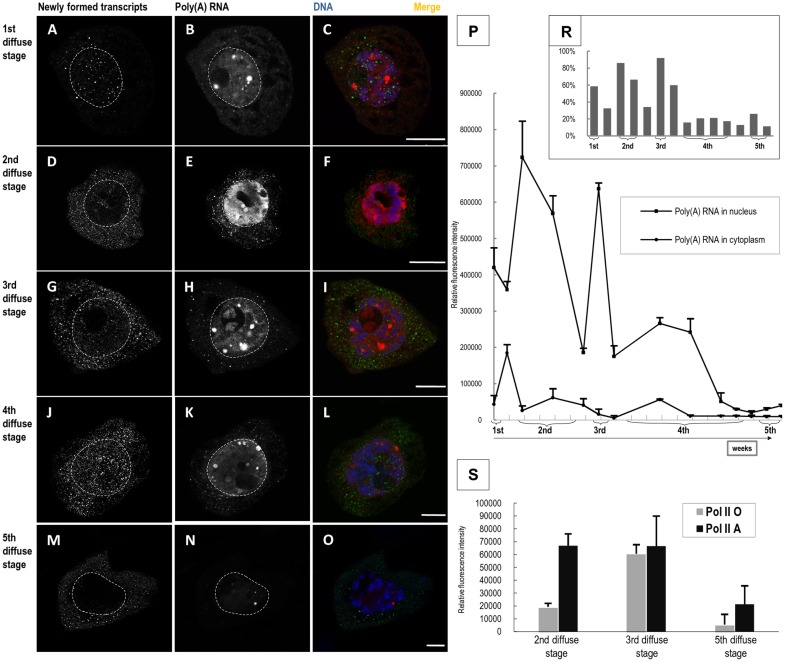
Poly(A) RNA distribution during all five diffuse stages of diplotene (A—O). Distribution of newly formed transcripts—BrU incorporation was performed with a long (90 min) incubation time (**A**, **D**, **G**, **J**, **M**). Distribution of poly(A) RNA during 5 diffuse stages (FISH with oligo d(T) probe) (**B**, **E**, **H**, **K**, **N**). Merge (**C**, **F**, **I**, **L**, **O**). Bars—10 μm. **Poly(A) RNA levels against background transcriptional activity in larch microsporocytes. P**—Poly(A) RNA level. 1st-5th—diffuse stages. **R**—Colocalisation between poly(A) RNA and newly formed transcripts. Arrows—contraction stages. **S—Level of both forms of RNA polymerase II: Pol II A and Pol II O during microsporocyte development** detected via immunostaining using the anti-phosph-serine5 CTD antibody (H14) and anti-phosph-serine2 CTD antibody (H5).

We also performed double labelling to examine the correlation between the distribution and levels of newly formed transcripts and poly-adenylated RNA (poly(A) RNA) (Fig. 3). Interestingly, the amount of nuclear poly(A) RNA was significantly higher than in the cytoplasm throughout the diplotene stage (Fig. 3B,3E,3H,3K and 3N). Nuclear poly(A) RNA was dispersed, and it also accumulated in numerous spherical structures that corresponded to Cajal bodies (CB) [24,28]. Cytoplasmic poly(A) RNA was mainly dispersed and sometimes appeared in small clusters (Fig. 3B,3E,3H,3K and 3N). We analysed the changes in poly(A) RNA levels in the nucleus and the cytoplasm. In the initial diplotene stages, the poly(A) RNA level was high and positively correlated with the level of newly formed transcripts. During these periods, we observed an intense signal in the nucleoplasm and CB (Fig. 3B,3E and 3H). In the fourth diffusion stage during the middle of diplotene, the poly(A) RNA level progressively decreased and remained low until the end of the diplotene stage (Fig. 3P). In the nucleus, we still observed poly(A) RNA in the dispersed form and in CBs, but the signal intensity was significantly lower (Fig. 3K and 3N).

We analysed the percentage of newly formed transcripts that were poly(A) RNAs. We performed double labelling with FISH immunofluorescence and measured the colocalisation of new transcripts with poly(A) RNA in the nucleus (Fig. 3R). We observed the highest level of nuclear poly(A) RNA during the second and third diffusion stages, which coincided with high transcriptional activity (Fig. 2M and Fig. 3P). During these stages, the percentages of poly(A) RNA colocalisation with newly formed transcripts corresponded to 86% and 92%, respectively, of the total newly formed RNA (Fig. 3R). In the stage with the highest colocalisation of newly formed transcript and poly(A) RNA, we also observed nucleolar poly(A) RNA localisation (Fig. 3H). Despite the high transcription level during the fourth diffusion stage, the nuclear poly(A) RNA level decreased compared to that of the previous periods of transcriptional activity; however, it was still quite high (Fig. 3P). The percentage of poly(A) RNA colocalisation with newly formed transcripts did not exceed 21% of the total poly(A) RNA during this period (Fig. 3P). These data suggest that the total poly(A) RNA \ in the fourth diffusion stage was primarily produced during synthesis in the earlier diffusion stages. The high level of newly formed transcripts in the second half of diplotene is most likely associated other RNA synthesis. During this period, newly formed transcripts accumulated in the nucleolus (Fig. 3J and 3L), and rRNA levels increased [25]. In the final stage of high transcriptional activity, the poly(A) RNA level was quite low, but as much as 26% of the total poly(A) RNA colocalised with newly formed transcripts (Fig. 3R).

We also examined RNA polymerase II levels associated with elongation (elongation form—Pol II O) and initiation of transcription (Pol II A) (Fig. 3S). During the second and third diffusion stages, the levels of these two polymerases were relatively high, coinciding with the period of intense poly(A) RNA synthesis. However, we observed a marked reduction in both hypo- and hyper-phosphorylated RNA polymerase II during the final diffusion stage.

### Splicing factor levels

These data indicate that mRNA is intensively synthesised, mainly during the early diplotene stages when RNA polymerase II levels are high. We investigated the level of uridine-rich small nuclear RNAs involved in splicing. Previous studies have shown that snRNPs undergo cyclic synthesis during early prophase [29]. We analysed the distribution and levels of mature snRNAs with a hypermethylated 5′-cap, including immunocytochemically localised m3G snRNAs and U2 snRNAs. We examined the total levels of both mature and immature RNAs and their localisation by using RNA FISH.

**Fig 4:**
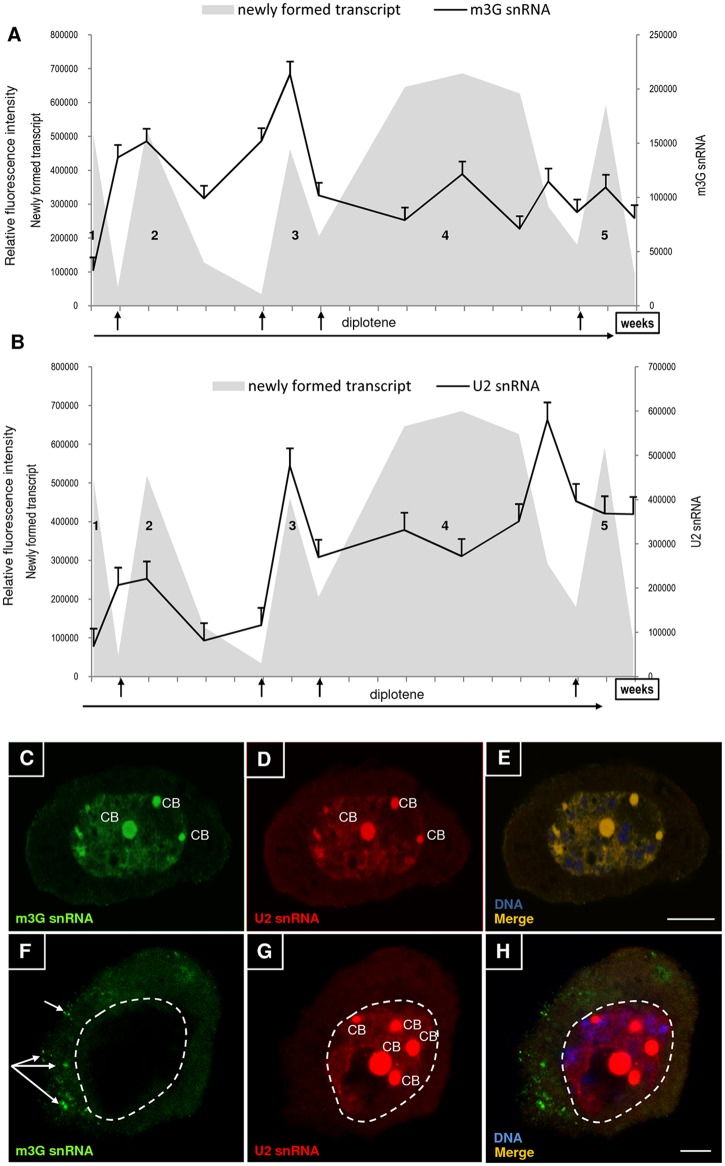
snRNA levels during microsporocyte development. **A**—mature snRNA (m3G snRNA) levels against background transcriptional activity; **B**—U2 snRNA levels relative to transcriptional activity; 1–5—diffuse stages. Arrows—contraction stages. **snRNA distribution during early and late diffusion diplotene stages. C**—distribution of U2 snRNA; **D**—distribution of m3G snRNA; **E**—merge. **F**—m3G snRNA primarily accumulates cytoplasmic clusters (arrows); **G**—U2 snRNA primarily accumulates in the nucleoplasm, forming numerous large Cajal bodies (CB); **H**—merge. Bar—10 μm.

The levels of mature snRNA and U2 snRNA underwent large fluctuations during the diplotene stage (Fig. 4A and 4B). Similar to the newly formed poly(A) RNA and RNA polymerase II transcripts, there were high levels of mature snRNA and U2 snRNA during the early diffusion diplotene stages, mainly the second and third (see Fig. 2M and Fig. 3P, 3S and Fig. 4A,4B). In the early diffuse stages, we observed nuclear snRNP localisation (Fig. 4C and 4D). Total and mature snRNA localisation overlapped with each other (Fig. 4E). In the second half of diplotene, when there was a decrease in poly(A) RNA synthesis, m3G snRNA levels remained moderate and exhibited little fluctuation (Fig. 4A). These short growth periods coincided with high transcriptional activity periods.

In contrast to mature snRNA, the level of U2 snRNA considerably increased in the second half of diplotene. We observed a biphasic increase in the U2 snRNA level: the first stage occurred at the beginning of the fourth diffusion stage, and the second and largest increase occurred at the end of the fourth diffusion stage. In the last diffusion stage, U2 snRNA decreased compared to that of the previous stage, although it remained high (Fig. 4B). During the final diplotene stages, we also observed a change in the distribution of these two forms of RNA. m3G snRNA primarily localised to the cytoplasm in the form of numerous clusters (Fig. 4F), whereas U2 snRNAs were primarily in the nucleoplasm, where they mainly accumulated in numerous CBs (Fig. 4G).

All control reactions produced negative results or very weak results compared to the standard reactions (S5 Fig.).

## Discussion

### Diffuse stages during meiotic division

Prophase of the first meiotic division is a period of dynamic chromosome behaviour [1]. After completion of crossing-over in pachytene and the synaptonemal complex breaks down, the chromosomes fully de synapse, except at the chiasmata. Early diplotene nuclei are filled with extended and fuzzy single chromosome threads, corresponding to the diffuse stage described in many organisms. This widespread phenomenon occurs in animal oogenesis but and spermatogenesis, as well during meiosis in many plant species [5,12,13,15,16,17,21]. However, the significance of diffusion stages isunknown, despite their frequent occurrence. This chromatin reorganisation suggests that the chromosomes become transcriptionally active. It has been suggested that the observed chromatin reorganisation correlates with cell growth processes at this stage of meiosis [21,22].

Research on chromatin morphology at this stage in *Larix leptolepis* has revealed profound changes in chromosome organisation, including four well documented diffuse substages: schisonema, pre-diffuse diplotene, mid-diffuse diplotene and post-diffuse diplotene [14]. The present study was performed on European larch microsporocytes, which are characterised by high synchronicity and a long diplotene stage that includes several diffusion stages that alternate with short contraction stages. These features allowed us to examine diffusion stages in terms of changes in chromatin morphology and activity.

### High transcriptional activity is characteristic of diffuse stages in larch microsporocytes

Our recent research has shown that the diplotene stage is the most transcriptionally active stage of prophase I. However, the level of newly formed transcripts during this long diplotene stage, which lasts approximately 5 months, fluctuates. We identified 5 periods of intense transcription separated by short periods of transcriptional silence. The periods of intense transcription in larch microsporocytes positively correlated with periods of chromatin decondensation, whereas during the chromatin contraction stages, transcription levels decreased. During insect spermatogenesis, the diffuse stage is associated with an intense and long period of cellular growth and important transcriptional activity [21,30]. Furthermore, findings in oocytes suggest a temporal relationship between chromatin remodelling and transcription activity [22]. Germinal vesicle (GV) bovine oocytes exhibit different patterns of chromatin configuration (GV0—period of chromatin decondensation, G1 to GV3—increase in chromatin condensation). The GV0 configuration exhibited high levels of RNA synthesis, the GV1 and GV2 stages showed a remarkable decrease in transcription, and the GV3 configuration was associated with a global repression in transcriptional activity. In interphase nuclei, it is generally accepted that transcriptional silencing occurs during chromatin condensation and that chromatin decondensation increases transcription [31]. Increases in the extent of chromosomal territory were observed only during RNA polymerase II transcription. Transcription inhibition (through AMD or DRB treatment) results in chromosome territories becoming more compact [32].

Our research revealed that the highest level of newly formed transcripts occurred during the 4th diffuse stage. At this stage, the chromatin consists of numerous fibres. Such chromatin organisation resembles the lampbrush chromosome (LBC). LBCs are found in the oocyte germinal vesicle of many vertebrate and invertebrate animals [33]. Immense amounts of RNA are synthesised and the oocyte volume increases several-fold when LBCs are present.

### Different RNA synthesis patterns during the diplotene stage in larch microsporocytes

Larch microsporocyte growth likely requires extensive protein synthesis. We determined the level of mRNA synthesis by analysing the levels of poly(A) RNA, RNA polymerase II (which is responsible for poly(A) RNA synthesis; [34,35]) and small nuclear RNAs (snRNAs, which are involved in mRNA maturation; [36–38]). We observed the highest level of poly(A) RNA during the first half of the diplotene stage. The level of poly(A) RNA positively correlated with the level of newly formed transcripts. During the second and third periods of high transcriptional activity, we observed a high degree of colocalisation between poly(A) RNA and newly formed transcripts, suggesting extensive mRNA synthesis.

These results were confirmed by the high content of hyperphosphorylated RNA polymerase II, which is responsible for pre-mRNA elongation, and the high level of snRNAs that are involved in transcript maturation. During the third period, a particularly high level of ribosomal RNA synthesis occurs in the nucleolus, which corresponds to an increase in cytoplasmic rRNA contents [25,28], reflecting the greater number of ribosomes necessary to carry out the increased protein synthesis. In the second half of diplotene, the transcriptional activity is maintained at a high level, although the level of total poly(A) RNA gradually decreases. The low level of poly(A) RNA colocalisation with newly formed transcripts and low RNA polymerase II content observed during the second half of diplotene suggest that mRNA synthesis is reduced. Other types of RNA are likely synthesised at the end of the diplotene stage. Previous studies have shown that during this period, larch microsporocytes synthesise significant amounts of rRNA [25,28]. In the fifth diffuse stage, phosphorylated RNA pol II signals significantly decreased but BrU staining still indicated high transcription levels. This small pool of active RNA polymerase II is likely sufficient to synthesise a small portion of the mRNAs measured during this period and for snRNA synthesis, which was synthesised and stored for subsequent developmental stages. However, the high BrU-RNA levels were likely related to RNA I and III polymerase activity. In the fifth diffuse stage, we observed high 26S rRNA and 5S rRNA levels (data not shown).

Interestingly, despite the low levels of newly formed mRNAs, snRNAs were maintained at high levels, particularly during the mid- and late diplotene stages. Larch microsporocytes likely accumulate and store snRNAs during late diplotene that will later be transmitted to the dyad cells during cell division (the half-life of snRNAs is long, often exceeding the cell life cycle) [39]. The large snRNA quantities that are synthesised and stored during the diplotene stage likely ensure that the cells contain the splicing machinery required during the development of a very transcriptionally active microspore [40–44]. Additionally, some snRNAs present in the larch microspore nucleus after cell division [45] are synthesised during the diplotene stage, and a high nuclear content of splicing factors has been observed in the early stages of *H*. *orientalis* microspores and *Brassica napus* [46–48]. The snRNA accumulation and transmission to dyads cells has also been observed in *Danio rerio* embryos, in which snRNAs are present in CBs at the 4-cell stage, which occurs 1 hour after fertilisation, and before zygotic genome activation at the 512-cell stage, which occurs approximately 2.75 hours after fertilisation [49].

The accumulation of snRNAs in diplotene microsporocytes may also be related to the maturation of these molecules. snRNP complex biogenesis (except for U6 snRNPs) is a complex process involving a specific sequence of events in the nucleus and cytoplasm. Following RNA polymerase II transcription, the original U snRNA transcripts are transported to the cytoplasm where they bind to Sm proteins, and the 5′-cap undergoes hypermethylation [29,50–52].snRNP complexes in the cytoplasm are re imported into the nucleoplasm. However, before new snRNPs can participate in pre-mRNA splicing, they are transported to CBs [38,53], where snRNA post-transcriptional modifications occur, including pseudouridylation and 2′-O-methylation [54–56].

By comparing changes in mature snRNA (m3G snRNAs with a hypermethylated 5′-cap structure) and U2 snRNA levels, considered here as total snRNAs, we found that their levels are quite different from each other. Total mature snRNA transcripts were maintained at a relatively constant level in the second half of diplotene, but total U2 snRNAs gradually increased. There was also a clear difference in their distribution. At the end of the diplotene stage, mature snRNA transcripts primarily localised to the cytoplasmin globular clusters. Similar structures known as cytoplasmic bodies are rich in snRNPs, and they have been observed in larch microsporocytes during premeiotic interphase and early prophase [29].

These structures most likely serve as a platform for snRNP complex assembly. The portion of U2 snRNA that did not colocalise with m3G snRNA primarily localised to the nucleoplasm, particularly in the large and numerous CBs present during this period. This lack of colocalisation suggests that the U2 snRNAs are in their immature form. The accumulation of immature U2 snRNAs in larch microsporocyte CBs may be associated with early snRNP biogenesis. Smith and Lawrence [57] previously reported the presence of pre-U2 snRNAs in HeLa cell CBs, and they suggested that U2 snRNA primary transcripts may be modified in CBs before being transported to the cytoplasm or that CBs may be involved in the RNA transport to the nucleolus where they are modified or to the pore complexes where they are transferred to the cytoplasm. The role of CBs in primary snRNA maturation (modifying the 3′-terminus) was confirmed in a recent study [58]. Studies performed in *Xenopus* oocytes have suggested that CBs also function as “supervisors” that control the correct assembly of a complex that exports snRNA to the cytoplasm and is composed of the CRM1 export receptor, CBC cap-binding complex and PHAX adapter [59]. Diplotene larch microsporocytes synthesise large quantities of snRNAs, and CBs likely serve as a storage/modification site in which RNAs wait for the assembly of the export complex and are then transmitted to the dyads.

### Summary

Our research shows that the diplotene stage in plants is a highly metabolically active stage. Our results contradict the view that the diffusion stage in larch cells is a resting period caused by low temperatures [2–4]. During this long period, microsporocytes increase their volume 3-fold, and this growth is associated with chromatin reorganisation and the occurrence of diffusion stages, which condition transcriptional activity. This mechanism is similar to that observed in oocytes, in which the diplotene stage is a period of intense growth and RNA synthesis [21,22,30,60–62]. However, in contrast to what occurs in oocytes, microsporocytes do not accumulate long-lived mRNA in the cytoplasm. Alternatively, mRNA is synthesised in large quantities in microsporocytes during early diplotene and is not transmitted to the dyad cells. However, at the end of the diplotene stage, snRNPs (splicing factors) are synthesised and accumulated, and they are likely transmitted to the dyads after division and subsequently to the tetrads, where they can then be used when intense transcription resumes.

## Supporting Information

S1 Fig Morphological changes in chromatin during diplotene part I. **1**—Chromatin in the form of small irregular patches, consisting of low density material; dense chromatin present in only a few small spots. **2**—Irregular patches of very dense chromatin; low-density chromatin was present only on the edge of patches. **3**—Large patches of closely packed chromatin; dense chromatin was primarily localised in the centre of each patch, and the less dense portion localised to the patch periphery. **4**—Large, elongated patches of chromatin surrounded by spaces of low chromatin content; dense chromatin primarily localised to the centre of each patch, and low-density chromatin localised to the edge of patches and penetrated into the free spaces in a fibrous form.
(TIF)

S2 Fig Morphological changes in chromatin during diplotene part II. **5–**2nd contraction stage—small compact patches of dense chromatin are separated by large spaces of low chromatin content; small amounts of less dense chromatin are visible on the edge of the patches. **6**–3rd diffuse stage—large, elongated patches primarily consisting of low-density chromatin. **7–**3rd contraction stage—irregular, compact chromatin patches primarily consisting of dense chromatin.
(TIF)

S3 Fig Morphological changes in chromatin during diplotene part III. **8—**During the first step of the 4th diffuse stage, chromatin was visible in the form of small, regular, closely spaced patches, composed primarily of low-density fibrils. **9**—At the second step of the 4th diffuse stage, much larger chromatin patches were observed, composed of delicate fibrils. **10**–During the third step of the 4th diffuse stage, chromatin was present in the form of large, regular patches separated by regions with low chromatin content. Within patches, dense chromatin localised to the centre of the patch and was surrounded by delicate chromatin fibrils. **11**—A fourth step of the 4th diffuse stage—chromatin is present in the form of large, elongated patches.
(TIF)

S4 Fig Morphological changes in chromatin during diplotene part IV. **12**–4th contraction stage—chromatin arranged in numerous, small, dense patches.**13**—a first step of the 5th diffuse stage—chromatin in the form of large, elongated, closely spaced patches consisting primarily of low-density chromatin. **14**—a second step of the 5th diffuse stage—chromatin arranged in the form of large, irregular patches separated by spaces with low chromatin content. Within patches, dense chromatin localised to the centre of the patch surrounded by delicate chromatin fibres.
(TIF)

S5 Fig Control reaction of double labelling of newly formed transcripts and poly(A) RNA. **A—**protoplasts without BrU incubation and treated with anti-BrU primary antibody and secondary antibody; **B**—hybridisation with sense oligo d(A) probe, **C**—merge. **Control reaction of double labelling of newly formed transcripts and pol II RNA. D**—protoplasts without BrU incubation and treated with anti-BrU primary antibody and secondary antibody; **E**—immunolocalisation reaction in the absence of pol II RNA-specific primary antibody (H5); **F**—merge. **Control reaction of the double labelling reaction of m3G snRNA and U2 snRNA. G**—immunolocalisation reaction in the absence of the m3G cap-specific primary antibody; **H**—hybridisation with the U2 snRNA sense probe; **I**—merge. Bars—10 μm.
(TIF)
