# Correction: Transcriptional Activity in Diplotene Larch Microsporocytes, with Emphasis on the Diffuse Stage

**DOI:** 10.1371/journal.pone.0125647

**Published:** 2015-04-22

**Authors:** The PLOS ONE Staff

There is an error in the third author’s name in the PDF version of the article. The correct name is: Michał Świdziński. The publisher apologizes for this error.

There is an error in the Supporting Information. S2 Fig, S3 Fig, and S4 Fig are all duplications of S1 Fig Please see the corrected S2 Fig, S3 Fig, and S4 Fig here.

## Supporting Information

S2 Fig Morphological changes in chromatin during diplotene part II. **5–**2nd contraction stage—small compact patches of dense chromatin are separated by large spaces of low chromatin content; small amounts of less dense chromatin are visible on the edge of the patches. **6**–3rd diffuse stage—large, elongated patches primarily consisting of low-density chromatin. **7–**3rd contraction stage—irregular, compact chromatin patches primarily consisting of dense chromatin.
(TIF)

S3 Fig Morphological changes in chromatin during diplotene part III. **8—**During the first step of the 4th diffuse stage, chromatin was visible in the form of small, regular, closely spaced patches, composed primarily of low-density fibrils. **9**—At the second step of the 4th diffuse stage, much larger chromatin patches were observed, composed of delicate fibrils. **10**–During the third step of the 4th diffuse stage, chromatin was present in the form of large, regular patches separated by regions with low chromatin content. Within patches, dense chromatin localised to the centre of the patch and was surrounded by delicate chromatin fibrils. **11**—A fourth step of the 4th diffuse stage—chromatin is present in the form of large, elongated patches.
(TIF)

S4 Fig Morphological changes in chromatin during diplotene part IV. **12**–4th contraction stage—chromatin arranged in numerous, small, dense patches.**13**—a first step of the 5th diffuse stage—chromatin in the form of large, elongated, closely spaced patches consisting primarily of low-density chromatin. **14**—a second step of the 5th diffuse stage—chromatin arranged in the form of large, irregular patches separated by spaces with low chromatin content. Within patches, dense chromatin localised to the centre of the patch surrounded by delicate chromatin fibres.
(TIF)
