# Supplementary figures and images for: Transcriptional Activity in Diplotene Larch Microsporocytes, with Emphasis on the Diffuse Stage
Source: PLoS One. 2015 Feb 11;10(2):e0117337. doi: 10.1371/journal.pone.0117337 (PMC4324999; doi:10.1371/journal.pone.0117337)

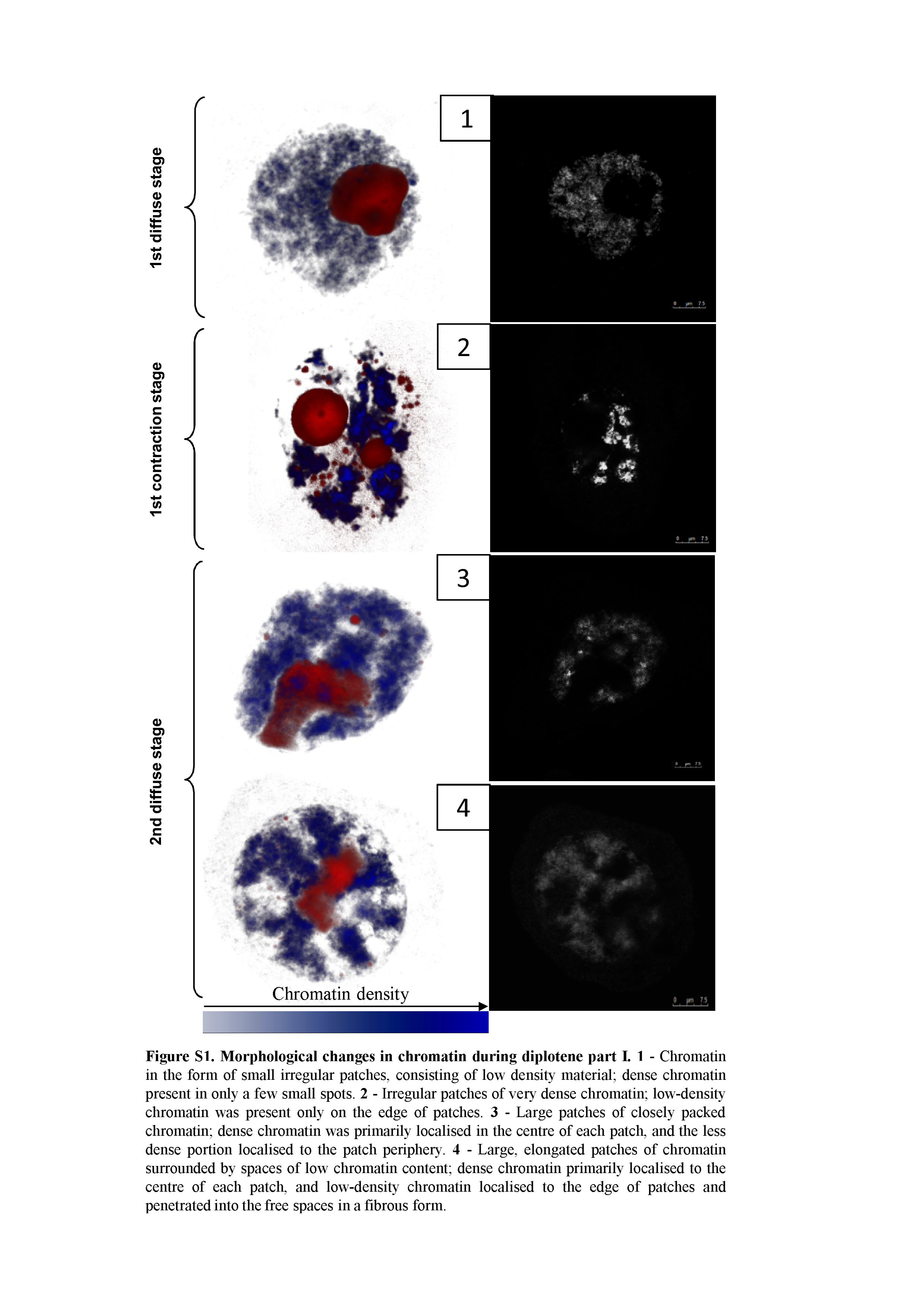

Supplement: S1 Fig — 1—Chromatin in the form of small irregular patches, consisting of low density material; dense chromatin present in only a few small spots. 2—Irregular patches of very dense chromatin; low-density chromatin was present only on the edge of patches. 3—Large patches of closely packed chromatin; dense chromatin was primarily localised in the centre of each patch, and the less dense portion localised to the patch periphery. 4—Large, elongated patches of chromatin surrounded by spaces of low chromatin content; dense chromatin primarily localised to the centre of each patch, and low-density chromatin localised to the edge of patches and penetrated into the free spaces in a fibrous form. (TIF) [file pone.0117337.s001.tif]

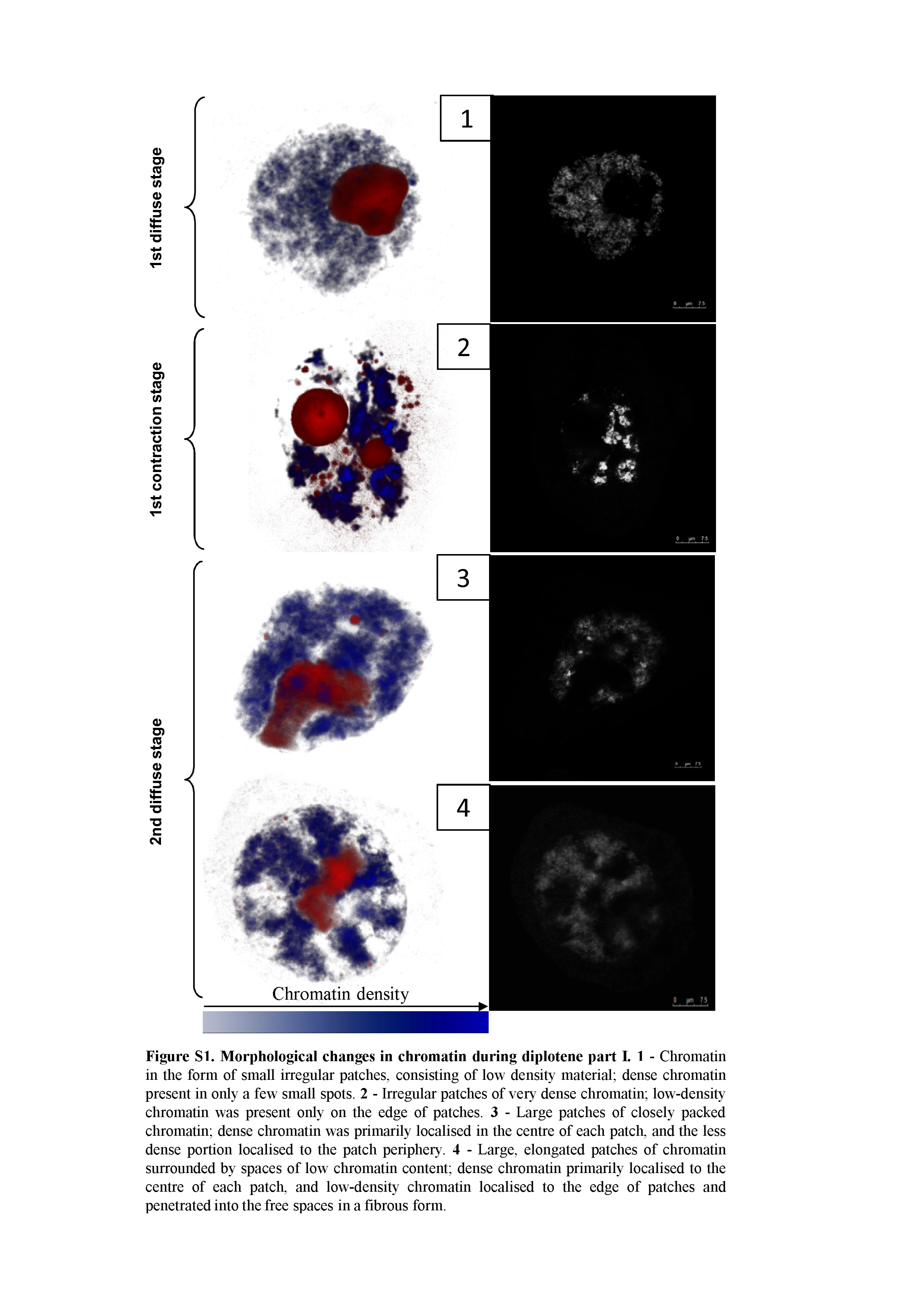

Supplement: S2 Fig — 5–2nd contraction stage—small compact patches of dense chromatin are separated by large spaces of low chromatin content; small amounts of less dense chromatin are visible on the edge of the patches. 6–3rd diffuse stage—large, elongated patches primarily consisting of low-density chromatin. 7–3rd contraction stage—irregular, compact chromatin patches primarily consisting of dense chromatin. (TIF) [file pone.0117337.s002.tif]

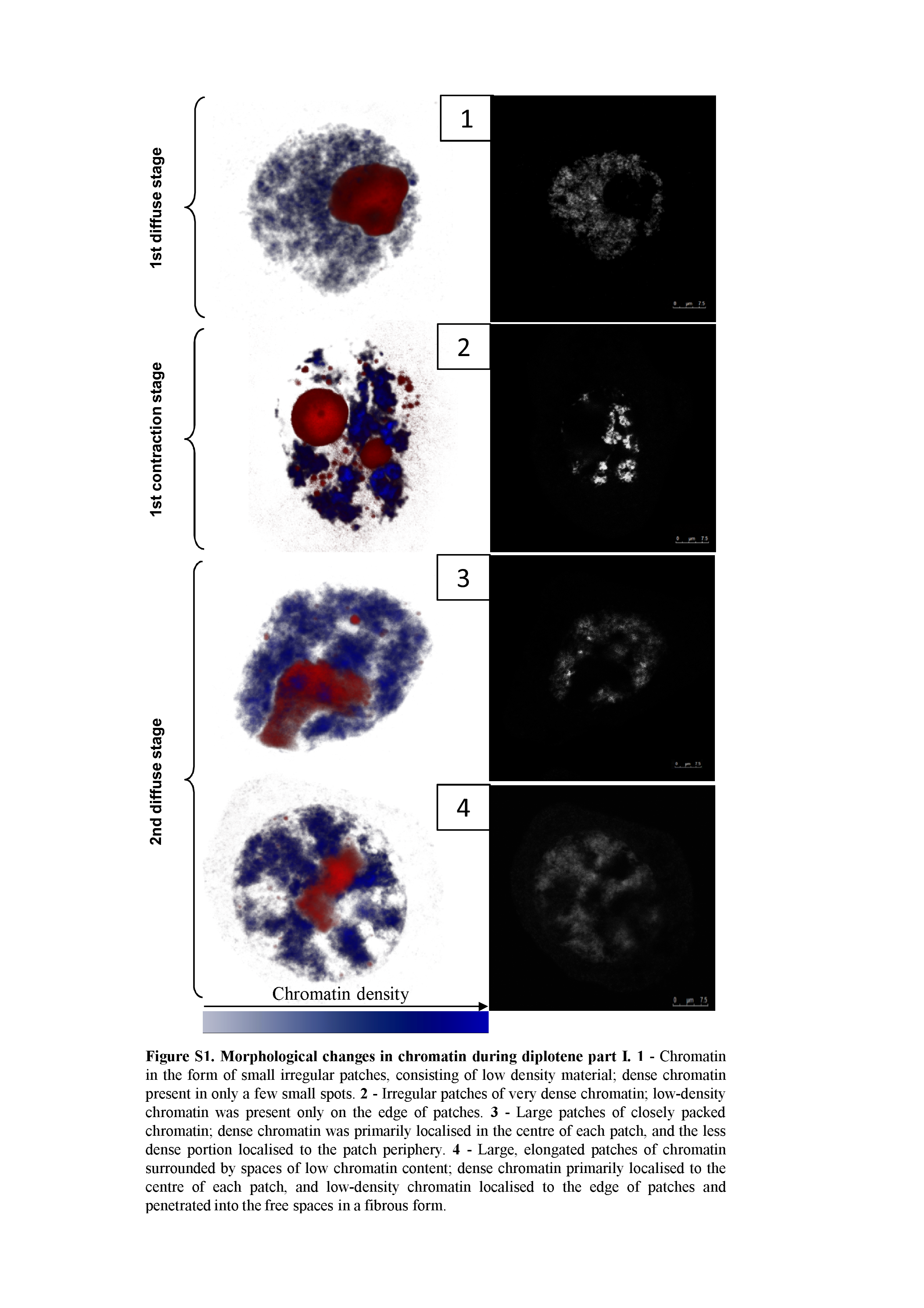

Supplement: S3 Fig — 8—During the first step of the 4th diffuse stage, chromatin was visible in the form of small, regular, closely spaced patches, composed primarily of low-density fibrils. 9—At the second step of the 4th diffuse stage, much larger chromatin patches were observed, composed of delicate fibrils. 10–During the third step of the 4th diffuse stage, chromatin was present in the form of large, regular patches separated by regions with low chromatin content. Within patches, dense chromatin localised to the centre of the patch and was surrounded by delicate chromatin fibrils. 11—A fourth step of the 4th diffuse stage—chromatin is present in the form of large, elongated patches. (TIF) [file pone.0117337.s003.tif]

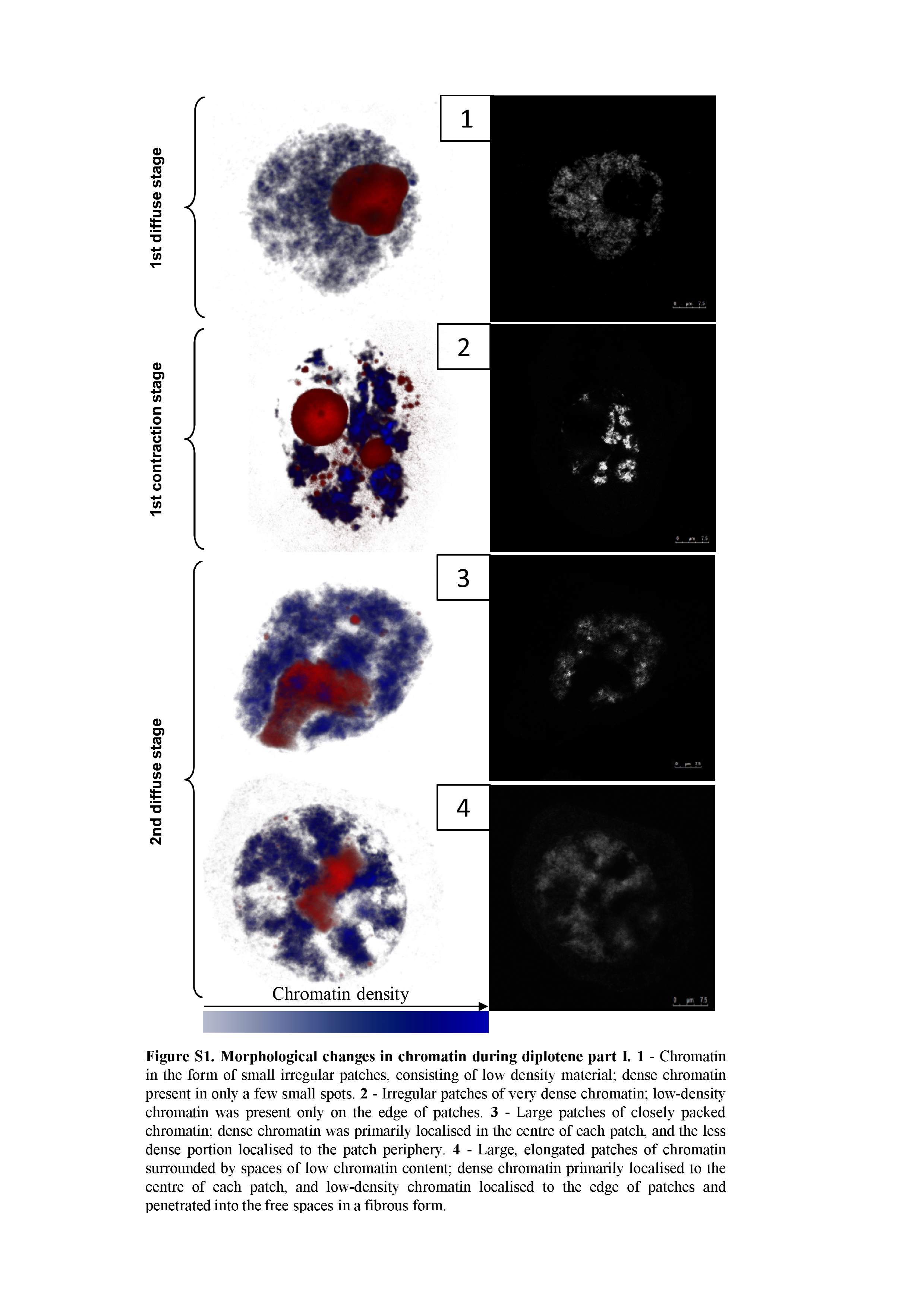

Supplement: S4 Fig — 12–4th contraction stage—chromatin arranged in numerous, small, dense patches.13—a first step of the 5th diffuse stage—chromatin in the form of large, elongated, closely spaced patches consisting primarily of low-density chromatin. 14—a second step of the 5th diffuse stage—chromatin arranged in the form of large, irregular patches separated by spaces with low chromatin content. Within patches, dense chromatin localised to the centre of the patch surrounded by delicate chromatin fibres. (TIF) [file pone.0117337.s004.tif]

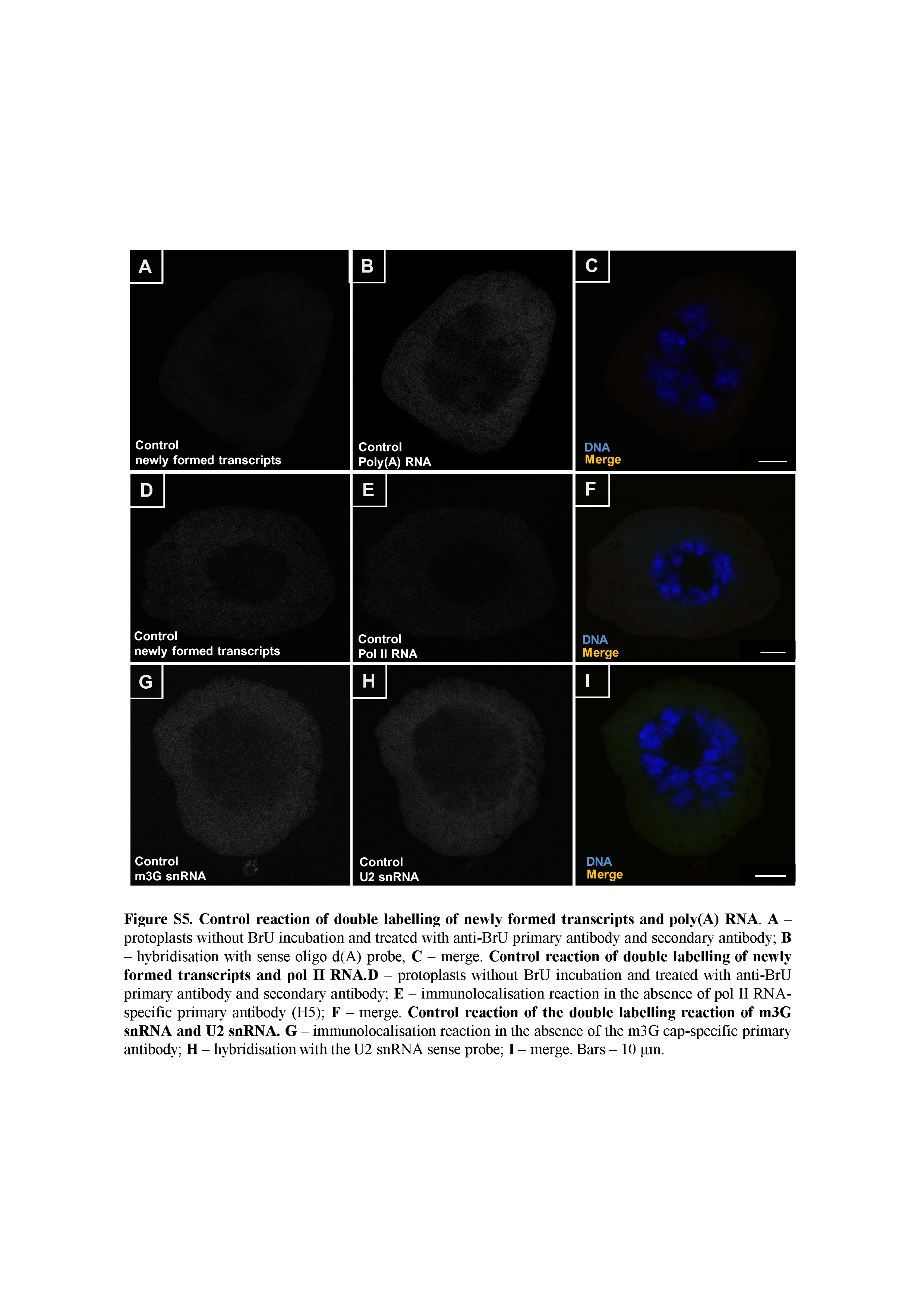

Supplement: S5 Fig — A—protoplasts without BrU incubation and treated with anti-BrU primary antibody and secondary antibody; B—hybridisation with sense oligo d(A) probe, C—merge. Control reaction of double labelling of newly formed transcripts and pol II RNA. D—protoplasts without BrU incubation and treated with anti-BrU primary antibody and secondary antibody; E—immunolocalisation reaction in the absence of pol II RNA-specific primary antibody (H5); F—merge. Control reaction of the double labelling reaction of m3G snRNA and U2 snRNA. G—immunolocalisation reaction in the absence of the m3G cap-specific primary antibody; H—hybridisation with the U2 snRNA sense probe; I—merge. Bars—10 μm. (TIF) [file pone.0117337.s005.tif]
