# Supplementary figures and images for: Correction: Transcriptional Activity in Diplotene Larch Microsporocytes, with Emphasis on the Diffuse Stage
Source: PLoS One. 2015 Apr 22;10(4):e0125647. doi: 10.1371/journal.pone.0125647 (PMC4406559; doi:10.1371/journal.pone.0125647)

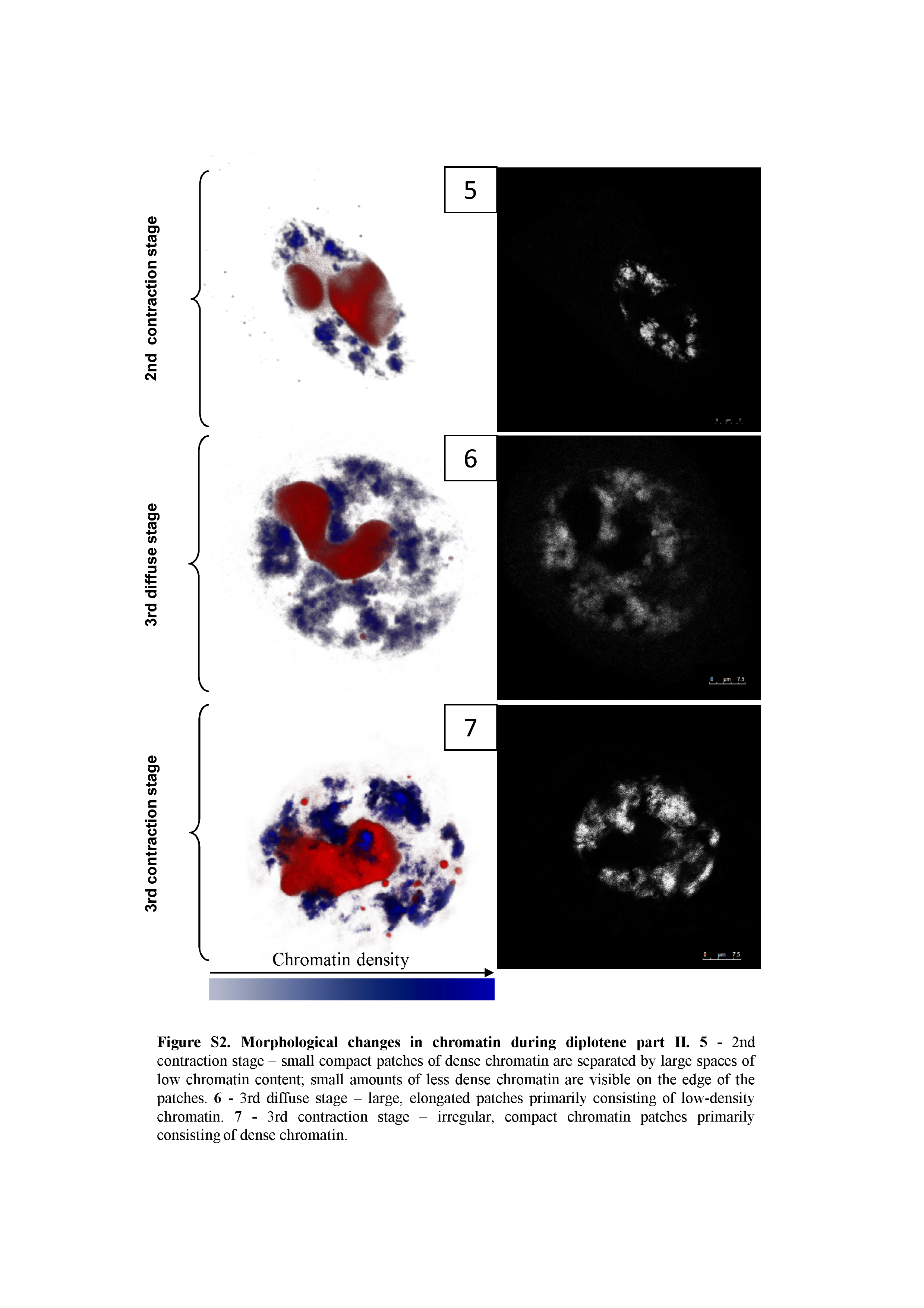

Supplement: S2 Fig — 5–2nd contraction stage—small compact patches of dense chromatin are separated by large spaces of low chromatin content; small amounts of less dense chromatin are visible on the edge of the patches. 6–3rd diffuse stage—large, elongated patches primarily consisting of low-density chromatin. 7–3rd contraction stage—irregular, compact chromatin patches primarily consisting of dense chromatin. (TIF) [file pone.0125647.s001.tif]

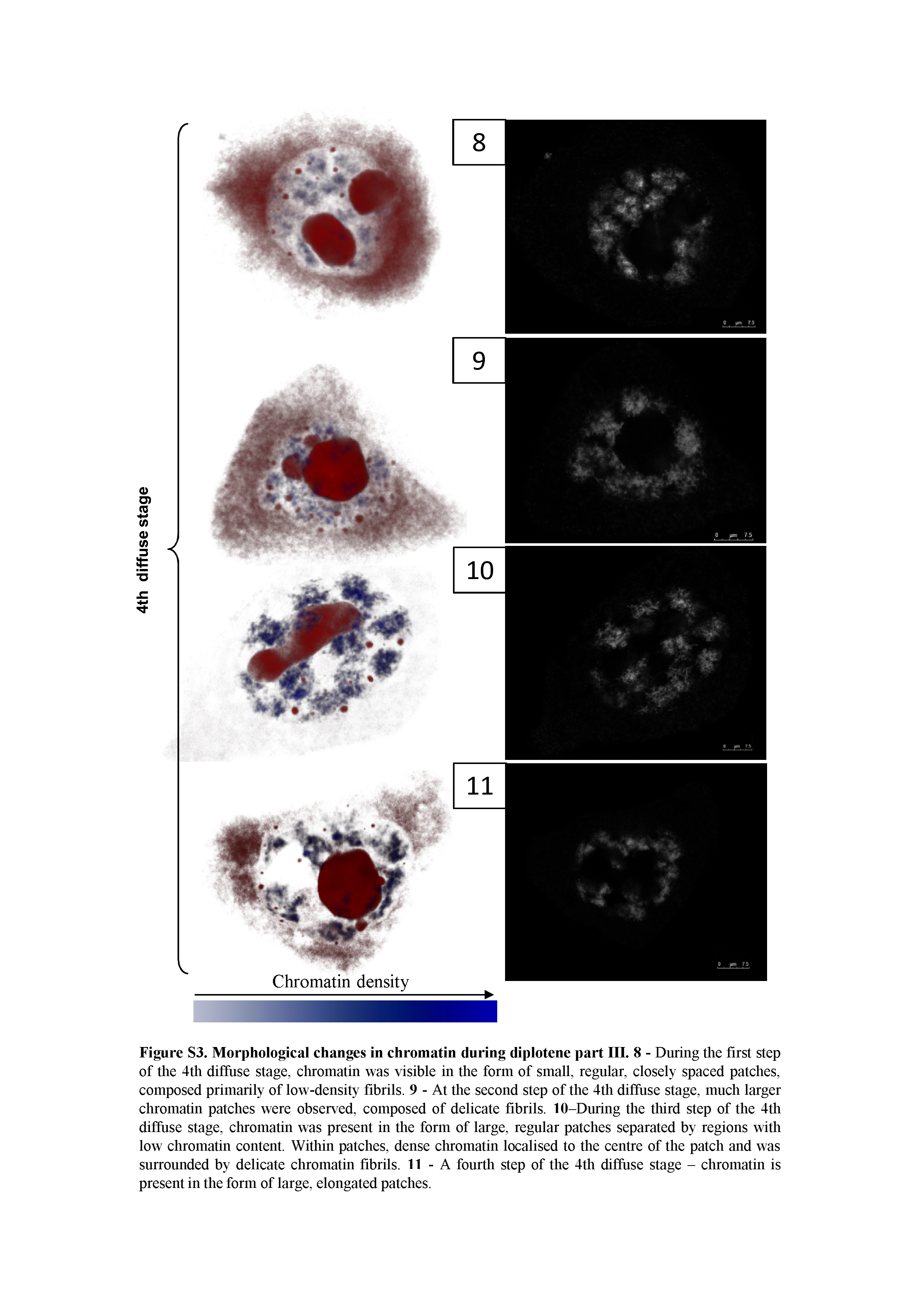

Supplement: S3 Fig — 8—During the first step of the 4th diffuse stage, chromatin was visible in the form of small, regular, closely spaced patches, composed primarily of low-density fibrils. 9—At the second step of the 4th diffuse stage, much larger chromatin patches were observed, composed of delicate fibrils. 10–During the third step of the 4th diffuse stage, chromatin was present in the form of large, regular patches separated by regions with low chromatin content. Within patches, dense chromatin localised to the centre of the patch and was surrounded by delicate chromatin fibrils. 11—A fourth step of the 4th diffuse stage—chromatin is present in the form of large, elongated patches. (TIF) [file pone.0125647.s002.tif]

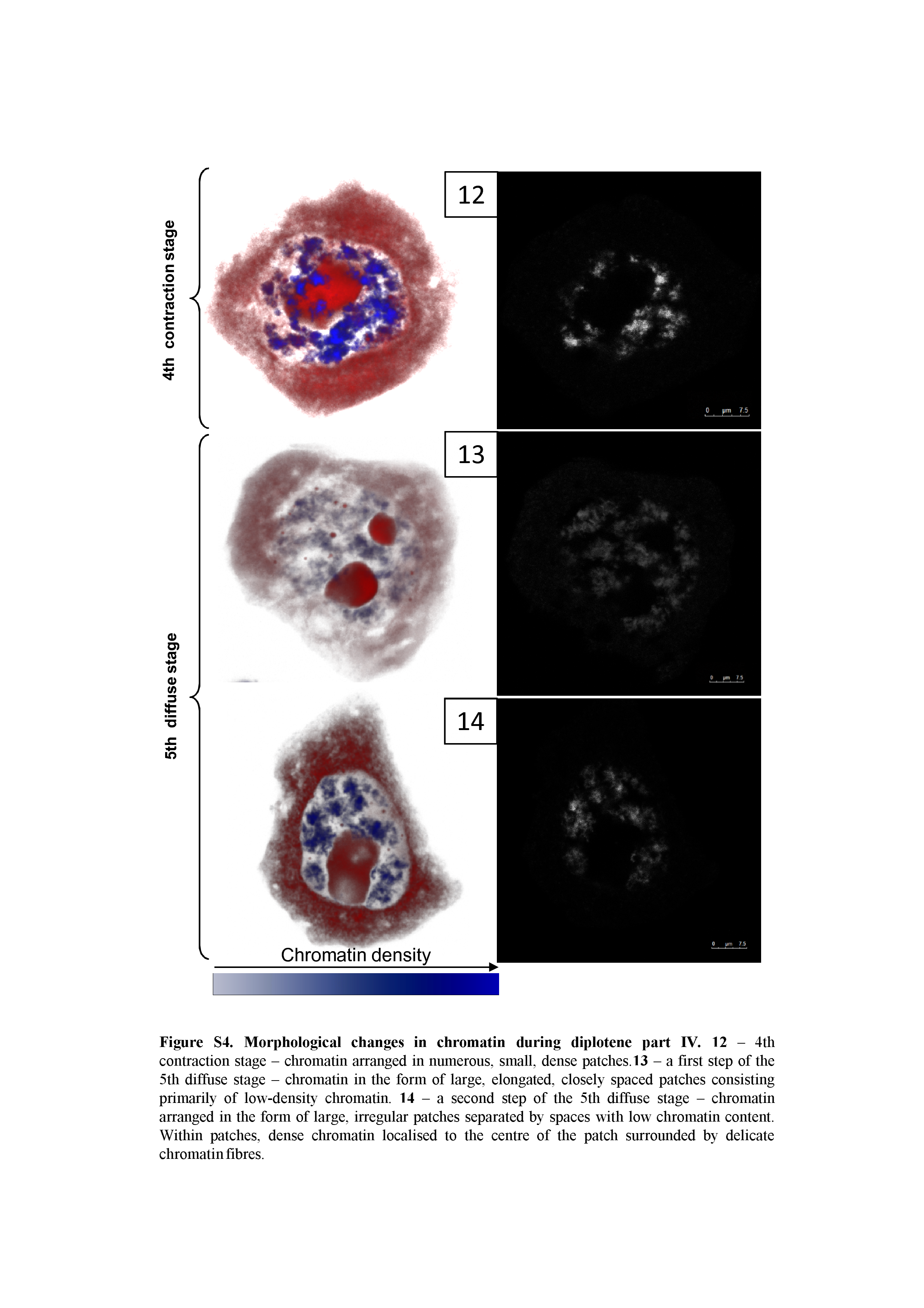

Supplement: S4 Fig — 12–4th contraction stage—chromatin arranged in numerous, small, dense patches.13—a first step of the 5th diffuse stage—chromatin in the form of large, elongated, closely spaced patches consisting primarily of low-density chromatin. 14—a second step of the 5th diffuse stage—chromatin arranged in the form of large, irregular patches separated by spaces with low chromatin content. Within patches, dense chromatin localised to the centre of the patch surrounded by delicate chromatin fibres. (TIF) [file pone.0125647.s003.tif]
